# Crime and subjective well-being in the countries of the former Soviet Union

**DOI:** 10.1186/s12889-015-2341-x

**Published:** 2015-10-03

**Authors:** Andrew Stickley, Ai Koyanagi, Bayard Roberts, Yevgeniy Goryakin, Martin McKee

**Affiliations:** The Centre for Health and Social Change, London School of Hygiene and Tropical Medicine, London, UK; The Stockholm Centre for Health and Social Change (SCOHOST), Södertörn University, 141 89 Huddinge, Sweden; Department of Human Ecology, Graduate School of Medicine, University of Tokyo, Tokyo, Japan; Research and Development Unit, Parc Sanitari Sant Joan de Déu, Fundació Sant Joan de Déu, Sant Boi de Llobregat, Barcelona, Spain; Instituto de Salud Carlos III, Centro de Investigación Biomédica en Red de Salud Mental, CIBERSAM, Spain; Health Economics Group, Norwich Medical School, University of East Anglia, Norwich, UK

**Keywords:** Former Soviet Union, Crime, Happiness, Life satisfaction, Subjective well-being

## Abstract

**Background:**

Criminal victimisation and subjective well-being have both been linked to health outcomes, although as yet, comparatively little is known about the relationship between these two phenomena. In this study we used data from nine countries of the former Soviet Union (fSU) to examine the association between different types of crime and subjective well-being.

**Methods:**

Data were obtained from 18,000 individuals aged 18 and above collected during the Health in Times of Transition (HITT) survey in 2010/11 in Armenia, Azerbaijan, Belarus, Georgia, Moldova, Kazakhstan, Kyrgyzstan, Russia and Ukraine. Information was obtained on respondents’ experience of crime (violence and theft) and self-reported affective (happiness) and cognitive (life satisfaction) well-being. Ordered probit and ordinary least squares (OLS) regression analyses were undertaken to examine the associations between these variables.

**Results:**

In pooled country analyses, experiencing violence was associated with significantly lower happiness and life satisfaction. Theft victimisation was associated with significantly reduced life satisfaction but not happiness. Among the individual countries, there was a more pronounced association between violent victimisation and reduced happiness in Kazakhstan and Moldova.

**Conclusions:**

The finding that criminal victimisation is linked to lower levels of subjective well-being highlights the importance of reducing crime in the fSU, and also of having effective support services in place for victims of crime to reduce its detrimental effects on health and well-being.

**Electronic supplementary material:**

The online version of this article (doi:10.1186/s12889-015-2341-x) contains supplementary material, which is available to authorized users.

## Background

The effects of crime impact across all levels of society [1]. At the national level, crime has been linked to lower economic growth [2] while generating large societal costs [3] through the operation of criminal justice and prison systems [1]. At the community level, it may stimulate and exacerbate the process of neighbourhood decline [4] by encouraging urban flight from cities perceived to have high crime rates [5]. Among individuals, criminal victimisation has been associated with a wide range of negative outcomes including worse physical and psychological health [6], economic losses as a consequence of lost earnings and the cost of medical care [1], and damage to intimate relationships [7]. Some evidence suggests that both crime victimisation and fear of crime might also result in poorer social, physical and occupational functioning among individuals [7, 8].

During the last decade, several studies have also linked being a victim of crime to differences in subjective well-being. Research on the emotional (happiness) and cognitive (life satisfaction) components of subjective well-being [9] undertaken in Europe [10, 11], Africa [12–14] and Asia [15] has shown decreased well-being in those experiencing violence, theft or burglary. However, this research has suggested that the relationship between crime and subjective well-being is complex. In particular, some studies have indicated that the strength of this relationship might vary in different parts of the world [16], that there are gender differences in the effects of some forms of crime on well-being [14], and that although crime does impact on subjective well-being, its effects are modest [17], especially when compared with other life events such as job loss [10, 15]. Indeed, a recent review of the effects of criminal victimisation on quality of life concluded that the association between victimisation and lower overall life satisfaction was ‘not robust’ [7].

Understanding how crime is associated with well-being is not only important in its own right but also because subjective well-being has been associated with a variety of outcomes. Longitudinal research has suggested, for example, that positive affect (happiness) precedes good relationships and productive and fulfilling work [18], while among healthy populations, subjective well-being has been linked to continued health and longevity [19]. Given this, it can be hypothesised that the effects of crime on health might not only be immediate and direct, but might also appear more gradually as a result of diminished well-being and its subsequent consequences, which among other things, may include an increased likelihood of engaging in risky health behaviours [20].

By extending research on the relationship between crime and subjective well-being to the former Soviet Union (fSU), this study sought to determine whether two forms of crime, violence and theft, were associated with subjective well-being (i.e. happiness and life satisfaction) in this setting and whether associations varied in the nine countries included in the study. There are several reasons to believe that this might be an important location to examine these relations. First, results from the International Crime Victims Survey (ICVS) have highlighted that although overall levels of victimisation in Western, Central and Eastern Europe, and the Commonwealth of Independent States are similar [21], the occurrence of crime differs markedly between the countries of the fSU. For example, reported victimisation rates are low in Azerbaijan (which has been linked to the more rural nature of that country [22]) but are much higher in countries such as Ukraine (although it is only a middle-ranking ICVS country in terms of the overall prevalence of victimisation) [23]. Differences in the occurrence of crime across these countries might be important as there is some evidence that the effects of crime on well-being might vary between regions according to the level of crime [13]. Second, not only are there differences in the occurrence of different types of crime such as assault and burglary within and between fSU countries [22], with some countries (e.g. Russia) having higher levels of extreme violence judging by the difference in homicide rates [24], but it is possible that the police response to crime might also differ across these countries. Although there is greater dissatisfaction in general with police performance in the ex-communist countries, recent research has suggested that there might be higher satisfaction in Georgia [22] whereas ‘predatory policing’ (with widespread police corruption and violence) has been reported in Russia [25]. Differences in police behaviour in the fSU countries might therefore mitigate or exacerbate the effects of victimisation on well-being. Third, research has already highlighted that crime seems to be having a detrimental effect on health in these countries as both concern about crime and criminal victimisation have been linked to greater psychological distress [26, 27] and worse self-rated health [27]. As other cross-country research has indicated that subjective well-being is especially low in Eastern Europe and fSU countries such as Russia [28, 29], it is possible that crime might be one of the factors underpinning this while also affecting public health as a result of diminished subjective well-being.

## Methods

### Study participants

Data were used from the Health in Times of Transition (HITT) survey. This was a cross-sectional survey undertaken in nine fSU countries. In 2010, data were collected in Armenia, Azerbaijan, Belarus, Georgia, Kazakhstan, Moldova, Russia, and Ukraine. However, because of political unrest, data collection was not undertaken in Kyrgyzstan until early 2011. In order to obtain nationally representative household samples, multi-stage random sampling was conducted in each country. Households were selected from within primary sampling units (approximately 100–200 per country) by the use of random route procedures. Within each selected household one adult aged 18 or above was randomly chosen to participate (determined by the nearest birthday). Information was collected by trained interviewers using a standard questionnaire, who conducted face-to-face interviews in the respondents’ homes. In every country, except in Russia and Belarus where Russian language was used, interviewees had the choice of responding in either their own country language or Russian.

In total, information was collected from 18,000 respondents. In six of the nine countries, the sample comprised 1800 respondents. However, in Russia and Ukraine, the sample sizes were larger (3000 persons and 2000 persons, respectively) in order to reflect these countries’ larger and more regionally diverse populations. The sample size was also larger in Georgia (*n* = 2200) following a booster survey of 400 additional interviews that was undertaken in late 2010 to ensure that the sample was more representative. Across the countries, response rates varied from 47 % (Kazakhstan) to 83 % (Georgia) [30].

### Ethical approval

Ethical approval was obtained from the London School of Hygiene and Tropical Medicine, and the survey was carried out in accordance with the Helsinki Declaration.

### Study variables

#### Dependent variables

To determine respondents’ level of subjective well-being, we used two measures commonly examined by researchers in this field i.e. *happiness* and *life satisfaction* [9]. For the former, respondents were asked “Taking all things together, how would you say things are these days – would you say you are?” and then presented with a single-item 10-point scale that ranged from ‘very unhappy’ (scored 1) to ‘very happy’ (scored 10). Using the same 10-point scale, information was obtained on life satisfaction by asking respondents, ‘How satisfied are you with your life as a whole?’ with answers ranging from ‘not satisfied at all’ (scored 1) to ‘extremely satisfied’ (scored 10).

#### Independent variables

Information was obtained on two forms of criminal victimisation. For *physical violence,* respondents were asked ‘During the past 12 months, have you been a victim of physical violence?’, while information on *theft* victimisation was obtained by asking respondents, ‘During the past 12 months, has anything been stolen from you?’ Both questions had ‘yes’ and ‘no’ answer options.

#### Control variables

A number of factors which have been associated with subjective well-being across countries [28] and which might be related to differences in criminal victimisation were adjusted for in the analysis. Respondents were categorised into three age groups 18–34, 35–59, and 60 and above. Marital status also comprised three categories: ‘married/cohabiting’, ‘never married’, and ‘divorced/widowed’. Educational level was classified as low (where respondents had less than complete secondary education), middle (complete secondary education), and high (incomplete or complete higher education). To assess respondents’ economic situation, information was collected on the possession of ten household assets. Principal component analysis was then used to generate wealth tertiles which were categorised as ‘high’, ‘average’ and ‘low’. The self-reported health status of respondents was categorised as either being ‘good/very good/fair’ or ‘poor/very poor’, while their residential location was categorised as being either ‘urban’ or ‘rural’. Finally, as previous research has shown that heavy episodic drinking is linked to criminal victimisation in the fSU [31], while higher alcohol consumption has been associated with reduced subjective well-being [32], we also adjusted for heavy episodic drinking in the analysis. Following the lead of a recent study [33], it was defined as consuming ≥2 l of beer, ≥750 g of wine, or ≥200 g of strong spirits in a single sitting. Details of all the variable questions are provided in Additional file 1.

### Statistical analyses

**Fig. 1:**
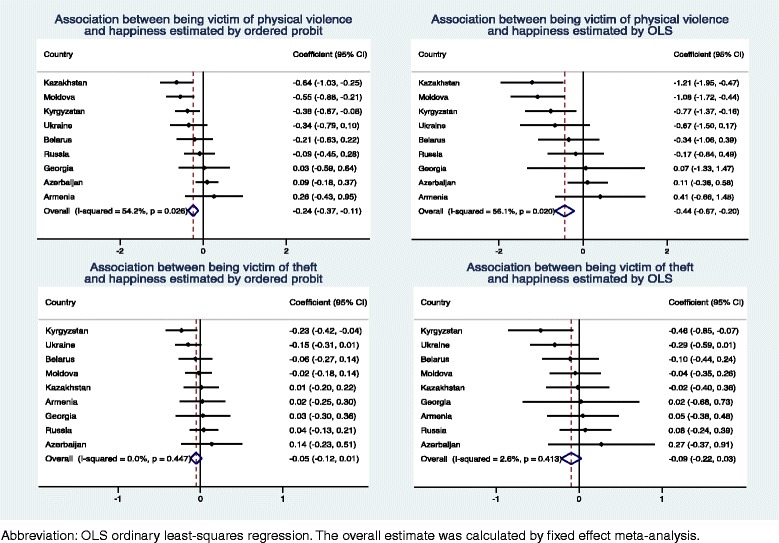
Association between criminal victimisation and happiness

**Fig. 2:**
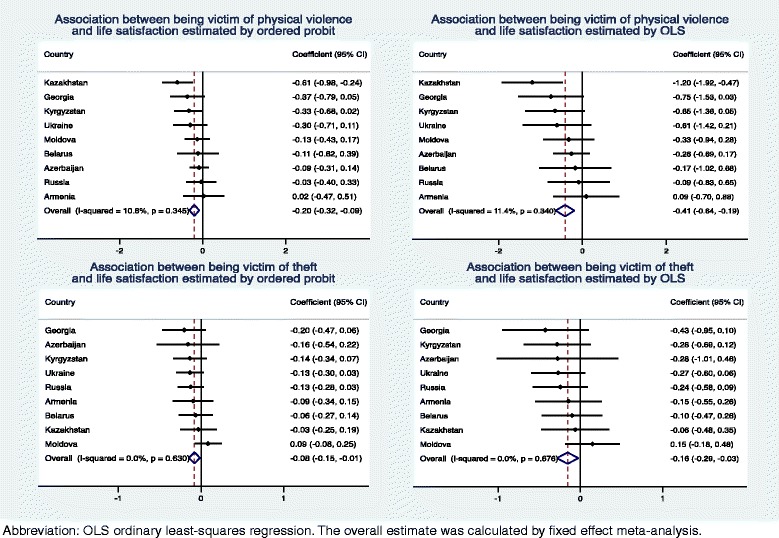
Association between criminal victimisation and life satisfaction

Details of the participants’ characteristics stratified by their experience of different forms of crime are presented in Table 1. Chi-square tests were used to determine if there were statistically significant differences. Although it has been suggested that in theory, ordered probit analysis should be more efficient for statistically analysing ordered outcomes such as those obtained from subjective well-being rating scales [34], in practice, there seems to be little difference in assuming either ordinality or cardinality of scores [35]. Given this, and following a recent recommendation [34], in this paper, the association between criminal victimisation and happiness and life satisfaction was assessed using both ordered probit and ordinary least squares (OLS) regression analysis as the results from the latter are more easily interpretable. The results from pooled country analyses using these statistical techniques are presented in Table 2. In addition, the country-wise estimates are also presented graphically in Figs. 1 and 2. The estimates for each country were combined into fixed-effect meta-analyses, with the Higgins’ I^2^ statistic being calculated. Higgins’ I^2^ corresponds to the degree of heterogeneity between countries that is not explained by sampling error. A <40 % heterogeneity is usually considered negligible, while 40–60 % indicates moderate heterogeneity [36]. For the analyses, β-coefficients and standard errors are presented (while 95 % confidence intervals are reported in the meta-analyses). The analyses combined males and females due to the low number of outcome events in some (female) categories. The statistical analysis was done with Stata 12.1 (Stata Corp LP, College station, Texas) with the level of statistical significance set at *p* < 0.05.

**Table 1:** Characteristics of the study sample by criminal victimisation status^a^

	Victim of physical violence^b^			Victim of theft^b^		
Characteristic	No	Yes	P-value	No	Yes	P-value
Age (years)						
18–34	6633 (98.0)	138 (2.0)	<0.001	6304 (93.4)	446 (6.6)	<0.001
35–59	7666 (98.7)	98 (1.3)		7335 (94.6)	416 (5.4)	
≥ 60	3370 (99.1)	29 (0.9)		3243 (95.6)	150 (4.4)	
Sex						
Male	7653 (98.1)	148 (1.9)	<0.001	7337 (94.2)	451 (5.8)	0.491
Female	10,016 (98.8)	117 (1.2)		9545 (94.4)	561 (5.6)	
Marital status						
Married/cohabiting	10,956 (98.8)	134 (1.2)	<0.001	10,514 (95.0)	559 (5.0)	<0.001
Never married	3591 (97.7)	85 (2.3)		3401 (92.8)	262 (7.2)	
Divorced/widowed	3059 (98.5)	46 (1.5)		2906 (93.9)	190 (6.1)	
Education^c^						
High	4858 (98.6)	69 (1.4)	0.030	4595 (93.6)	315 (6.4)	0.026
Middle	10,479 (98.6)	147 (1.4)		10,035 (94.6)	568 (5.4)	
Low	2288 (97.9)	49 (2.1)		2210 (94.6)	127 (5.4)	
Wealth^d^						
High	5536 (98.6)	79 (1.4)	0.376	5208 (93.1)	383 (6.9)	<0.001
Average	6249 (98.6)	87 (1.4)		5983 (94.7)	337 (5.3)	
Low	5884 (98.3)	99 (1.7)		5691 (95.1)	292 (4.9)	
Self-rated health						
Good/fair	14,375 (98.6)	200 (1.4)	0.011	13,755 (94.6)	788 (5.4)	0.007
Poor	3240 (98.0)	65 (2.0)		3080 (93.4)	218 (6.6)	
Location						
Urban	10,658 (98.5)	161 (1.5)	0.886	10,147 (94.1)	633 (5.9)	0.123
Rural	7011 (98.5)	104 (1.5)		6735 (94.7)	379 (5.3)	
Heavy episodic drinking^e^						
No	15,631 (98.7)	205 (1.3)	<0.001	14,950 (94.6)	846 (5.4)	<0.001
Yes	2038 (97.1)	60 (2.9)		1932 (92.1)	166 (7.9)	
Country						
Armenia	1782 (99.3)	13 (0.7)	<0.001	1723 (96.2)	68 (3.8)	<0.001
Azerbaijan	1753 (98.1)	34 (1.9)		1734 (97.5)	45 (2.5)	
Belarus	1784 (99.2)	15 (0.8)		1696 (94.2)	104 (5.8)	
Georgia	2188 (99.5)	11 (0.5)		2141 (97.5)	54 (2.5)	
Kazakhstan	1770 (98.3)	30 (1.7)		1678 (93.6)	114 (6.4)	
Kyrgyzstan	1757 (97.8)	39 (2.2)		1687 (94.1)	105 (5.9)	
Moldova	1749 (97.4)	46 (2.6)		1614 (89.9)	181 (10.1)	
Russia	2939 (98.7)	39 (1.3)		2801 (94.6)	160 (5.4)	
Ukraine	1947 (98.1)	38 (1.9)		1808 (90.9)	181 (9.1)	

^a^Data in numbers (percentages)

^b^Refers to events which occurred in the past 12 months

^c^Education was classified as: low (less than complete secondary education), middle (complete secondary education), high (incomplete or complete higher education)

^d^Principal component analysis was used to generate a wealth index based on the possession of ten household assets

^e^Heavy episodic drinking was defined as consumption of at least one of the following on one occasion: ≥2 l of beer, ≥750 g of wine, or ≥200 g of strong spirits

**Table 2:** Association between criminal victimisation and happiness or life satisfaction

	Happiness^a^				Life satisfaction^b^			
Explanatory variables	Ordered probit		OLS		Ordered probit		OLS	
Victim of physical violence^c^	−0.2956***		−0.5589***		−0.2325***		−0.4687***	
	(0.0687)		(0.1297)		(0.0650)		(0.1287)	
Victim of theft^c^		−0.0412		−0.0834		−0.0693*		−0.1388*
		(0.0351)		(0.0652)		(0.0345)		(0.0682)
Age (years)								
18–34	ref.	ref.	ref.	ref.	ref.	ref.	ref.	ref.
35–59	−0.2453***	−0.2440***	−0.4558***	−0.4535***	−0.2334***	−0.2319***	−0.4651***	−0.4619***
	(0.0200)	(0.0201)	(0.0368)	(0.0368)	(0.0198)	(0.0199)	(0.0392)	(0.0392)
≥ 60	−0.2032***	−0.1979***	−0.3812***	−0.3715***	−0.0966***	−0.0935**	−0.1956***	−0.1893***
	(0.0285)	(0.0286)	(0.0532)	(0.0533)	(0.0285)	(0.0285)	(0.0564)	(0.0565)
Sex								
Male	ref.	ref.	ref.	ref.	ref.	ref.	ref.	ref.
Female	0.0592***	0.0609***	0.1081***	0.1115***	0.0027	0.0041	0.0089	0.0115
	(0.0167)	(0.0167)	(0.0310)	(0.0310)	(0.0167)	(0.0167)	(0.0331)	(0.0331)
Marital status								
Married/cohabiting	ref.	ref.	ref.	ref.	ref.	ref.	ref.	ref.
Never married	−0.1028***	−0.1035***	−0.1856***	−0.1866***	0.0284	0.0297	0.0511	0.0539
	(0.0223)	(0.0223)	(0.0411)	(0.0412)	(0.0225)	(0.0226)	(0.0444)	(0.0445)
Divorced/widowed	−0.3291***	−0.3292***	−0.6276***	−0.6281***	−0.2364***	−0.2352***	−0.4760***	−0.4734***
	(0.0247)	(0.0248)	(0.0465)	(0.0466)	(0.0238)	(0.0239)	(0.0474)	(0.0474)
Education^d^								
High	ref.	ref.	ref.	ref.	ref.	ref.	ref.	ref.
Middle	−0.0778***	−0.0780***	−0.1478***	−0.1482***	−0.1708***	−0.1716***	−0.3397***	−0.3415***
	(0.0185)	(0.0185)	(0.0341)	(0.0342)	(0.0187)	(0.0187)	(0.0367)	(0.0367)
Low	−0.1105***	−0.1124***	−0.2107***	−0.2140***	−0.1743***	−0.1753***	−0.3506***	−0.3527***
	(0.0297)	(0.0297)	(0.0551)	(0.0551)	(0.0293)	(0.0293)	(0.0579)	(0.0579)
Wealth^e^								
High	ref.	ref.	ref.	ref.	ref.	ref.	ref.	ref.
Average	−0.2198***	−0.2191***	−0.4103***	−0.4093***	−0.2451***	−0.2459***	−0.4895***	−0.4910***
	(0.0199)	(0.0199)	(0.0366)	(0.0366)	(0.0202)	(0.0202)	(0.0397)	(0.0397)
Low	−0.4329***	−0.4349***	−0.8107***	−0.8152***	−0.4339***	−0.4368***	−0.8660***	−0.8715***
	(0.0223)	(0.0224)	(0.0411)	(0.0412)	(0.0227)	(0.0227)	(0.0445)	(0.0445)
Self-rated health								
Good/fair	ref.	ref.	ref.	ref.	ref.	ref.	ref.	ref.
Poor	−0.4347***	−0.4375***	−0.8328***	−0.8387***	−0.5184***	−0.5201***	−1.0492***	−1.0527***
	(0.0244)	(0.0245)	(0.0460)	(0.0462)	(0.0238)	(0.0239)	(0.0471)	(0.0472)
Location								
Urban	ref.	ref.	ref.	ref.	ref.	ref.	ref.	ref.
Rural	0.0812***	0.0825***	0.1530***	0.1554***	0.0467**	0.0473**	0.0932**	0.0942**
	(0.0173)	(0.0174)	(0.0323)	(0.0323)	(0.0173)	(0.0173)	(0.0342)	(0.0342)
Heavy episodic drinking^f^								
No	ref.	ref.	ref.	ref.	ref.	ref.	ref.	ref.
Yes	−0.0899***	−0.0914***	−0.1670***	−0.1700***	−0.0771**	−0.0787**	−0.1533**	−0.1563**
	(0.0250)	(0.0250)	(0.0467)	(0.0466)	(0.0257)	(0.0257)	(0.0509)	(0.0510)

*OLS* ordinary least-squares regression, *ref.* reference category

Data are coefficient (SE). All analyses are adjusted for country

^a^Happiness was assessed by the question “Taking all things together, how would you say things are these days – would you say you are?” with answers provided on a 10-point scale that ranged from ‘very unhappy’ (scored 1) to ‘very happy ‘(scored 10)

^b^Life satisfaction was assessed by the question “How satisfied are you with your life as a whole?” with answers provided on a 10-point scale that ranged from ‘not at all satisfied ‘(scored 1) to ‘extremely satisfied ‘(scored 10)

^c^Refers to events which occurred in the past 12 months

^d^Education was classified as: low (less than complete secondary education), middle (complete secondary education), high (incomplete or complete higher education)

^e^Principal component analysis was used to generate a wealth index based on the possession of 10 household assets

^f^Heavy episodic drinking was defined as consumption of at least one of the following on one occasion: ≥2 l of beer, ≥750 g of wine, or ≥200 g of strong spirits

*p < 0.05, **p < 0.01, ***p < 0.001

## Results

The characteristics of the study sample by criminal victimisation status are presented in Table 1. Respondents who were younger, never married, had poor self-rated health, and who engaged in heavy episodic drinking had a significantly higher likelihood of experiencing both forms of criminal victimisation. The prevalence (%) of violent crime was highest in Moldova and Kyrgyzstan, while for theft it was in Moldova and Ukraine.

The results of the analyses examining the association between criminal victimisation and happiness and life satisfaction are shown in Table 2. Experiencing physical violence was significantly associated with lower happiness scores and reduced life satisfaction in both the probit and OLS regression analyses. Theft was significantly associated with reduced life satisfaction but not lower happiness. Most of the results for the other variables were consistent across outcome categories and type of analysis. Thus, being older, divorced/widowed, having less education, wealth, being in poorer health, a heavy episodic drinker and living in an urban location were all associated with significantly lower subjective well-being scores. Interestingly, although women were significantly happier than men, there was no difference in terms of their life satisfaction scores.

The impact of violence and theft victimisation on subjective well-being in the individual countries is presented in Figs. 1 and 2. In both the probit and OLS analyses, there was statistically significant between-country heterogeneity for the association between being a victim of violence and happiness with the effect being most pronounced in Kazakhstan and Moldova (Higgins′ I^2^ 54.2–56.1 %), while a weaker but statistically significant association was also observed in Kyrgyzstan. For other associations, there was no statistically significant between-country heterogeneity.

## Discussion

This study examined the association between criminal victimisation and subjective well-being (i.e. happiness and life satisfaction) in nine countries of the fSU. In pooled country analyses, having been a victim of violence was associated with significantly reduced happiness and life satisfaction scores whereas theft was associated only with lower life satisfaction. In terms of the individual countries, violent victimisation was associated with reduced happiness very strongly in Kazakhstan and Moldova.

The finding that victimisation was associated with lower subjective well-being in the fSU accords with findings from other parts of the world [10–15]. However, earlier research also raised questions about the importance of victimisation for well-being with some studies suggesting that its effects were either very small [17] or overshadowed by the impact of other phenomena such as poverty [37] and unemployment [10, 15, 37]. A recent study which compared the size of the coefficients from a regression analysis when examining the association between victimisation and subjective well-being in 20 sub-Saharan African countries, found that although crime victimisation was negatively and significantly associated with well-being, unemployment had approximately four and 1.5 times the effect of theft and physical assault victimisation respectively [14]. When making the same type of comparison, our results suggest that the impact of victimisation is also modest in the fSU countries, as being in poor health and having a low level of wealth both had a stronger effect on subjective well-being than physical violence, while many variables were more important for well-being than being a victim of theft.

Besides the direct effects of physical injury and/or psychological trauma, it is possible that victimisation might affect subjective well-being in a number of ways. Janoff-Bulman and Frieze have suggested, for example, that experiencing crime can destroy an individual’s basic assumptions about themselves and the world, resulting in both high levels of stress and anxiety [38] and possibly from this study’s perspective, lower levels of happiness and life satisfaction. Alternatively, the experience of crime and victimisation has been linked to changes in behaviour and lifestyle such as staying in at night, or changing residence or workplace [37, 38], which in conjunction with crime’s detrimental effects on other life domains, may impact negatively on a victim’s overall quality of life [7] and result in diminished feelings of subjective well-being.

In the individual country analyses, experiencing violent victimisation had a strong impact on happiness in Kazakhstan and Moldova and to a lesser extent in Kyrgyzstan. After Russia, these countries have the highest mortality rates from interpersonal violence in the World Health Organisation’s (WHO) European Region [39] and there is some evidence that levels of (unreported) non-lethal violence might also be very high [40]. In such an environment victimisation might be affecting well-being in different ways. Earlier research from the ICVS has highlighted for example, that citizens in Kazakhstan and Kyrgyzstan are among the most nervous going out after dark [21]. If personal safety concerns (either as a result of one’s own or others’ victimisation) is restricting behaviour, then it might be leading to deterioration in the overall quality of life, while our own earlier research in the fSU countries has linked concern about crime to an increased risk of psychological distress [26].

It is also possible that other factors might be important for the low levels of subjective well-being among victims of violence in these particular countries such as not being able to get help and support after experiencing violence. Earlier research undertaken in the fSU revealed that many women who experience intimate partner violence do not tell anyone about it for a range of reasons including its perceived normality, embarrassment or because they think that it would “not do any good” [41]. A recent report from Kazakhstan has further highlighted that even when domestic abuse is reported to the police, many complaints are subsequently withdrawn for a variety of reasons including financial and family pressure [40]. As earlier research has indicated that perceived social support (such as the perceived availability of guidance and emotional support) is especially important for the psychological health of victims of violent crime [42], then being unable to discuss abuse might be extremely detrimental for well-being. Moreover, it might possibly help explain why in Moldova, where the largest number of female respondents in the current study reported experiencing violence, and where many women do not discuss their experience of abuse with anyone [41], violent victimisation was associated with reduced happiness.

There are several limitations to this study. As the data were cross-sectional, we could not determine the direction of the observed relationships between the variables. One earlier study has suggested that victims of crime are unhappier than non-victims even before being victimised and that being less satisfied with life might itself be associated with an increased likelihood of victimisation [43]. Also, we cannot discount the possibility that individuals who are less satisfied or unhappier might have interpreted or reported crime differently from other people. In addition, even though some research suggests that different types of crime might impact differently on subjective well-being among males and females [14], we were unable to stratify the analyses by sex due to the low number of self-reported crimes among some participants in some countries. Further, the questions concerning victimisation were crude in the sense that they provided no information on the actual form of the event, where it occurred, who was involved, how long it lasted etc. In future research more detailed information should be obtained about the crimes people experience as this may further elucidate the relationship between criminal victimisation and subjective well-being. It should also be acknowledged that there were many factors that could have affected the association between victmisation and subjective well-being that we were unable to take into account in the analysis, including the quality of the judicial system, personal insurance, the availability and quality of medical care, and whether there were victim compensation schemes in place.

## Conclusions

This study has shown that experiencing different forms of crime such as violence and theft has a detrimental effect on subjective well-being in the countries of the fSU. As subjective well-being is itself increasingly being recognised as a predictor of future health outcomes, it is possible that the effects of crime might impact on health over a longer period of time as a result of diminished subjective well-being and its consequences. Regardless of the specific mechanisms involved, the finding that crime diminishes subjective well-being reinforces the need not only to reduce crime in the countries of the fSU, but also, to have effective support services in place to counter the effects of crime on victims’ well-being and health.

## Additional file

Additional file 1:
**Variable questions.** (DOCX 16 kb)
