# Supplementary material for: Crime and subjective well-being in the countries of the former Soviet Union
Source: BMC Public Health. 2015 Oct 3;15:1010. doi: 10.1186/s12889-015-2341-x (PMC4592744; doi:10.1186/s12889-015-2341-x)
Supplement: Additional file 1: — Variable questions. (DOCX 16 kb) [file 12889_2015_2341_MOESM1_ESM.docx]

**Appendix 1**

Variable questions

Dependent variables

**Taking all things together, how would you say things are these days – would you say you are?**

*Use a 10-point scale, where 10 means that you are absolutely happy, and 1 absolutely unhappy.*

**How satisfied are you with your life as a whole?**

*Use a ten-point scale, where 10 means that you are completely satisfied, and 1 – completely dissatisfied.*

Independent variables

**During the past 12 months, have you been a victim of physical violence?**

| 1 = Yes |
| --- |
| 2= No |
| 99 = Refused to answer  **During the past 12 months, has anything been stolen from you?**   \| 1 = Yes \| 98 = Don’t know \| \| --- \| --- \| \| 2 = No \| 99 = Refuse to answer \| |

Control Variables

**Respondent’s gender**

| 1 = Male | 2 = Female |
| --- | --- |

**What is your age? (***Write the number of complete years***)**

__________Years

**What is your marital status?**

| 1 = Have never been married | 4 = Widow/Widower – within the last 5 years |
| --- | --- |
| 2 = Married/co-habiting | 5 = Widow/widower – for more than 5 year |
| 3 = Divorced | 99 = Refusal |

**What is your highest level of education?**

| 1 = Primary or without education | 5 = Non-finished higher education |
| --- | --- |
| 2 = Incomplete secondary (basic) | 6 = Completed higher education |
| 3 =Completed secondary education (including vocational) | 99 = Refusal |
| 4 = Completed secondary special (college) |  |

**Which of the following things does the household own?**

*Interviewer, multiple responses are allowed*

1 = Fridge

2 = TV

3 = Washing machine (not automatic)

4 = Mobile telephone

5 = Computer

6 = Car produced since 2005

7 = Automatic washing machine

8 = Home cinema

9 = Dishwasher

10 = Car made since 2005

11 = None of the above

98 = Don’t know

99 = Refuse to answer

**In general would you say your health is…**

1 = Very good 98 = Don’t know

2 = Good 99 = Refuse to answer

3 = Fair

4 = Poor

5 = Very poor

**Type of living location**

1 = Capital of the country

2 = Regional capital

3 = City (but not country or regional capital)

4 = Settlement of urban type

5 = Village

**How much strong spirits such as vodka do you usually drink on one occasion?**

1 = Don’t know

_______grams 2 = Refused to answer

**How much beer do you usually drink on one occasion (in litres or shares of a liter)?**

1 = Don’t know

_______litres 2 = Refused to answer

**How much industrially produced wine/Champaign do you usually drink on one occasion?**

1 = Don’t know

_______litres 2 = Refused to answer
